# Porcelain Inlays

**Published:** 1906-04

**Authors:** A. W. Dana

**Affiliations:** Burlington, Iowa.


### PORCELAIN INLAYS.

By A. W. Dana, D. D. S., Burlington, Iowa.

The question that naturally arises when the subject is being investigated is: Do I need porcelain in my practice? The answer is a most emphatic affirmation. Why is this so?

If you are striving for higher levels in your professional career there is no one thing that will help more to elevate than becoming proficient in the use of porcelain. It demands that the user must (and I speak knowingly) believe that cleanliness is next to godliness. In fact, I will almost say it takes preference. One of the most necessary requisites in the successful manipulation of porcelain is patience. If you have it not naturally there is to my mind nothing that will develop it so perfectly as the effort to master this subject. With the development of these two points, cleanliness and patience, you can not help but acquire stick-too-itiveness and care of detail. While our work should all tend to perfection in these points it is possible to conduct a practice without doing so, and much too often this is the case. With porcelain it is an impossibility to reach even that stage of proficiency where we can deceive the public into believing they are being well served and succeed.

There is an unlooked for reward in perfecting yourself in porcelain which comes unconsciously. You find the detail of cavity preparation for gold and amalgam fillings as well as the manipulation of the gold in building fillings has become much easier. And you have developed a delicacy of touch that assists wonderfully in handling with good results, that bugbear, treatment and filling of root canals.

What is to be gained from the general public by using porcelain material?

Undoubtedly it brings to your practice persons who appreciate the esthetic, and being appreciative are willing to compensate for the higher skill called to play. It brings you in touch with people holding high ideals, with a purpose in life, pleasant to be with, inspiring in us a desire to make the most of the opportunities presented outside of business, often tending to financial gain by good investments.

The hours at the office are shortened, not at the cost of the income; and life is more livable in every way.

To me these points seem sufficient incentives to cause any man to take up the use of porcelain.

From another point of view the dentist who fails to use the new material is certainly not only standing still but is losing ground rapidly. There is a time coming, gentlemen, and it is no distant day, when the majority of cavities in a wide-awake practice will be filled by inlay porcelain and gold. And by so filling cavities, dentists will increase their usefulness to mankind.

The one who is successful as a gold operator you will find is a man working with a system. He has certain fundamental principles applying to cavity preparations, certain fixed principles for manipulating gold foil and finishing fillings. He adheres to these as closely as the individual case will permit, always keeping in mind the ideal. I have found the most successful porcelain men are the ones who have a system in their work. Cavity preparations are worked out on a mechanical basis. Adapting the matrix, building the inlay, in fact, all steps are methodically carried to completion.

Nothing is done in a hit-or-miss manner. Such a little matter apparently as mixing the cement used to seat the inlay receives their most careful attention. This systematic care of detail runs through the entire operations and insures success.

The question of what bodies to use is one much debated. The only real objection to the low fusing bodies that I have is their opaqueness, while the high fusing bodies give a more translucent effect, the inlay looking more lifelike. It is not so much a question of what body you use as it is, are you master of that body? This in all its phases under varying conditions. If so, good results will follow, and vice versa.

To become master of any particular subject means each step must be taken in hand and thoroughly understood.

In inlays numerous operations must be performed in each step on dummies in the laboratory.

In the slightly biscuited technique teeth furnished us by the dental depots, cavities can be formed until you have mastered cavity formation.

After baking the teeth and mounting them in wood blocks so they are easily handled, platinum is adapted to these cavities time and again until the idiosyncracies of platinum are fully grasped. Bodies are mixed and the inlays built in layers until after repeated effort carving, contouring, baking, shrinkage and securing proper shades, are fully mastered in every detail. Keep this up persistently, going from the simple cavities to the more difficult ones. And it is not a far cry to the practical inlay, giving the patient so many of the requisites of the ideal filling, namely, blending in harmoniously with the surrounding tissues a perfect non-conductor and the most perfect substitute for lost tooth structure so far known to man.

Don't, for porcelain's sake, gentlemen, become cranks on this subject, but use it always when indicated.

Some men use inlays successfully in cavities where other men make flat failures. Learn your limitations and govern yourself accordingly.

Porcelain inlays should be used always in labial and approximal cavities in the six anterior teeth. Also in most all cavities in these teeth where the incisal is involved, being careful to have the incisal margin carried to a point where the strain is at a minimum and always secure as much bulk as the case will permit for strength. In bicuspids and molars porcelain is only demanded when the esthetic outweighs utility. Gold inlays in all bicuspids and molars are preferable in cavities except buccal.

